# Plasmapheresis in thyrotoxicosis: a single-center case series

**DOI:** 10.1186/s13256-024-04480-9

**Published:** 2024-03-30

**Authors:** I. Rami, D. Zerrouki, I. Assarrar, S. Rouf, H. Latrech

**Affiliations:** 1 Department of Endocrinology-Diabetology and Nutrition, Faculty of Medicine and Pharmacy, Mohammed VI University Hospital Center, University of Mohammed 1st, 4806, 60049 Oujda, Morocco; 2 Laboratory of Epidemiology, Clinical Research and Public Health, Faculty of Medicine and Pharmacy, Mohammed VI University Hospital Center, University of Mohammed 1st, 4806, 60049 Oujda, Morocco

**Keywords:** Plasmapheresis, Thyrotoxicosis, Anti-thyroid agents, Drug-induced abnormalities, Case report

## Abstract

**Background:**

Plasmapheresis represent an alternative therapeutic option for hyperthyroidism with thyroid storm or refractory cases. It provides a rapid decrease in plasma thyroid hormones and anti-thyroid antibodies. The aim of this paper was to report our single center’s experience in managing particular situations of hyperthyroidism using apheresis.

**Cases presentation:**

The following case series describes three young African patients (two females, one male) aged 29, 37, and 25 years old, respectively, with Graves’ disease who presented with drug ineffectiveness, drug-induced agranulocytosis, and thyroid storm with multi-organ failure. The three patients underwent plasmapheresis sessions leading to effective decline of thyroid hormone levels and offering a window for processing total thyroidectomy.

**Discussion/conclusion:**

The standard management of thyrotoxicosis and thyroid storm was usually codified by the concomitant use of antithyroid medication, iodine, beta-blockers, and corticosteroids. This medical preparation can be effective in most cases. However, drug toxicity or ineffectiveness can limit the use of such therapeutics. Our paper supports the efficiency and safety of therapeutic plasma exchange in the preoperative management of thyrotoxicosis.

## Introduction

Thyrotoxicosis is a clinical syndrome characterized by the excess of circulating thyroid hormones. In most instances, the excess comes from increased production by the thyroid gland. In this case, it may be caused by Graves’ disease (GD), toxic multinodular goiter (TMNG), and toxic nodules (TN) [1].

Management strategies include reducing thyroid hormone synthesis and release, inhibiting the conversion of thyroxin (T4) to triodothyronine (T3), and moderating the peripheral effects of excess thyroid hormone [2, 3]. Therefore, there are different options for treatment depending on the etiology. Thionamides are the first line of treatment in the majority of patients with hyperthyroidism period. Other options are radioactive iodine and thyroid surgery [4].

In patients with severe hyperthyroidism, further therapies are needed in the acute phase to restore the euthyroid status, therapies such as potassium iodide, beta-adrenergic receptor blockers, glucocorticoids, and therapeutic plasma exchange (TPE). Therapeutic plasma exchange is an alternative treatment that was introduced in the 1970s for hyperthyroidism management [5]. TPE is an extracorporeal blood purification method considered to remove large-molecular-weight substances bound to plasma proteins such as pathogenic auto-antibodies, immunocomplexes, cryoglobulins, cholesterol-containing lipoproteins, and plasma-protein-bound thyroid hormones. Albumin and fresh frozen plasma (FFP) are used as replacement fluids in TPE for thyrotoxic patients [6].

The indications of TPE for thyrotoxic patients may join category II of apheresis indications, as determined by the American Society for Apheresis (ASFA) [7]. Nevertheless, there was formerly no clear consensus recommendation for or against its use in patients with hyperthyroidism without thyroid storm [7].

The effectiveness of the treatment is determined by the volume of blood being processed, the volume of the plasma exchanged in each process, the frequency of exchange, and other technical features. However, as with any invasive process, TPE also has side effects [8, 9].

We report in this series, three cases of thyrotoxic patients who required the use of plasma exchanges. The aim of our work was to study the effectiveness and safety of this therapeutic approach in the preoperative management of thyrotoxic patients.

## Case presentation

### Case 1

A 25-year-old African man was admitted to the emergency room (ER) for worsening palpitations, and asthenia, without chest pain or dyspnea. He was diagnosed with Graves’ disease 1 year before, with poor compliance with carbimazole and propranolol therapy.

He stopped taking his medication 1 month after diagnosis. A total of 1 year later, he presented with systolic heart failure with dilated cardiomyopathy. He weaned from alcohol and drug abuse 5 years ago. The patient was treated with spironolactone 50 mg/day, propranolol 40 mg twice daily, furosemide 40 mg/day, ramipril 12.5 mg/day, and digoxin 0.25 mg/day.

On initial evaluation, the heart rate was 125 beats per minute (bpm), blood pressure (BP) was at 110/60 mmHg, his temperature was at 36.5°C, respiratory rate 24 breaths/minute, and SaO_2_ was 100%. Physical examination found a skinny man who was discreetly agitated but alert and oriented to place and time with a bilateral proptosis. The patient also presented with a non-active Graves’ orbitopathy. We especially noted signs of heart failure, manifested by jugular venous distension, bilateral lower-extremity edema, and hepatomegaly. There were bibasilar crackles over the lungs. Cervical palpation found a symmetric diffusely enlarged and firm thyroid gland.

The electrocardiogram (ECG) showed atrial fibrillation and poor R wave progression with a rate of 130 bpm. Chest X-ray showed mild cardiomegaly. Laboratory investigations showed severe thyrotoxicosis with a free thyroxine (FT4) at 500 pmol/l [normal values (NV): 12–22]. There were also cholestasis and impaired liver function tests. However, liver and cardiac enzymes were not elevated (Table 1).

**Table 1:** Laboratory findings during the course of the first patient’s illness (Case 1)

Tests	Day 1**	Day 3*	Day 4*	Day 5*	Day 6	Day 18	Normal range
TSHus (mUI/L)	< 0.05						0.25–4.2
FT4 (pmol/l)	500	500	100	75	45	8	12–22
WBC (µ/l)	6520	4490	7190	5210	4290	6610	7000–10,000
ANC (µ/l)	3320	1970	3490	2220	2680	5210	3000–5000
AST (UI/l)	45	56	49	62	75	60	5–45
ALT (UI/l)	26	28	26	32	33	27	5–35
CRP (mg/l)	36.9	32.7	23.7	25.6	22.7	21	0–5
Calcemia (mg/l)	85	83	85	93	93	84	84–105
PT (%)	40	46	50	48	50	50	70–100

*WBC* white blood cells, *ANC* absolute neutrophils count, *PT* prothrombin time, *AST* aspartate aminotransferase, *ALT* alanine aminotransferase, *CRP* C-reactive protein, *TSH* thyroid-stimulating hormone, *TSHus* ultrasensitive TSH assay, *FT4* free thyroxine

*Days of plasmapheresis

**Admission in ICU

Regarding his liver dysfunction, he benefited from a complementary workup that included negative viral serologies, as well as a liver ultrasound that showed liver damage secondary to heart failure without lesions or abnormalities in the bile ducts.

Transthoracic echocardiogram showed a biventricular dilated cardiomyopathy, with an ejection fraction of less than 34% and global hypokinesis. Pericardial effusion of 3 mm was found too. In addition, he had a pleural effusion objectified on a chest computed tomography (CT) scan. The removed fluid was transudative. Cervical ultrasound noted a voluminous multinodular goiter with nodules classified as EU-TIRADS3, and a thyroid volume at 100 cc.

The patient was transferred to the cardiology intensive care unit (ICU).

He received cardiology resuscitation. He was kept on the same heart medications with propranolol dose optimization to 40 mg three times a day. He was then transferred to the Endocrinology–Diabetology and Nutrition department after stabilization of his heart condition.

In our department, the patient was started on iodide potassium upon admission, and then prednisone was added on the eighth day of his transfer. In view of his deteriorating status and our inability to start on carbimazole owing to worsening liver dysfunction and his heart failure, a decision was made to begin TPE, taking into account the excessive level of FT4 (Table 1).

In total the patient received three sessions of TPE over 3 consecutive days, each with 2.5 L of FFP.

After the last plasmapheresis session, there was an exceptional decline in levels of FT4 and transaminases (Table 1). Iodide potassium was continued for 14 days, while propranolol was retained until clinical and biological euthyroidism was obtained. Total thyroidectomy was recommended once hyperthyroidism was controlled. However, the patient refused surgery and discontinued all medication. A total of 1 month later, the patient was admitted to the ER for severe arrhythmia and died on the second day of admission.

### Case 2

A 37-year-old African woman was admitted to the ER for fever, chills, mucositis, sore throat, angina, and generalized body aches. She had been diagnosed with Graves’ disease 2 months before admission. She was put on methimazole (15 mg/day) and propranolol (80 mg/day) with good adherence.

On initial evaluation, the temperature was at 39 °C, heart rate was at 105 bpm, BP was at 90/54 mmHg, respiratory rate 25 breaths/minute, and SaO_2_ was at 100%. The physical examination was unremarkable except for homogeneous thyroid hypertrophy and proptosis. Blood workup showed an undetectable TSH, significantly elevated levels of free T4, and agranulocytosis (Table 2).

**Table 2:** Laboratory findings during the course of the second patient’s illness (Case 2)

Test	Day 1 (admission in ICU)	Day 10*	Day 14	Day 20**	Day 21**	Day 22***	Normal range
TSHus (mUI/L)	< 0.05	< 0.05					0.25–4.2
FT4 (pmol/l)	67	91	85.7	33.5	23.5	19.32	12–22
TRAb (U/l)		20					< 1.75
WBC (µ/l)	1130	3320	4430	5100	6240	9670	7000–10,000
ANC (µ/l)	110	1510	1450	2540	3000	7490	3000–5000
AST (UI/L)	36	35	31	24	23	20	5–45
ALT (UI/L)	13	15	11	12	10	10	5–35
CRP (mg/l)	307	1.19	0.77	1.46	7.85	15.29	0–5
Calcemia (mg/l	96	96	93	88	92	89	84–105
PT (%)	88%			92	90	100	70–100
Hb (g/dl)	10.9			11			
Hct (%)	32			35			
MCV (Fl)	77			78			

*WBC* white blood cells, *ANC* absolute neutrophils count, *PT* prothrombin time, *AST* aspartate aminotransferase, *ALT* alanine aminotransferase, *CRP* C-reactive protein, *TSH* thyroid-stimulating hormone, *TSHus* ultrasensitive TSH assay, *FT4* free thyroxine, *TRAb* thyrotrophin receptor antibody, *Hb* hemoglobin, *Hct* hematocrit, *MCV* mean corpuscular volume

*Day of admission in the Department of Endocrinology

**Days of TPE

***Evaluation before surgery

Clinical and biological evaluation in this patient resulted in a diagnosis of septic shock secondary to methimazole-induced agranulocytosis (Table 2). Therefore, methimazole was stopped, and the patient was admitted to the ICU. She received standard resuscitation measures and antibiotic therapy for urinary tract infection.

The patient improved her absolute neutrophil count (ANC) and inflammatory markers. Table 2 summarizes the results of the first blood workup and follow-up.

The transthoracic echocardiogram was normal, and the cervical ultrasound showed a homogeneous goiter measuring 25 ml in volume.

After stabilization, the patient was transferred to our department to manage the thyrotoxicosis condition. She was put on propranolol 40 mg three times a day (on day 10), prednisolone 60 mg per day (on day 14), potassium iodide 20 drops orally every 8 hour, and cholestyramine 4 g three times a day. A total of 10 days later, we noted a good clinical course. Nevertheless, thyroid hormone levels were still increased (Table 2).

Given the agranulocytosis episode and consistently excessive thyroid hormone levels, TPE was highly recommended in this case.

On hospital day 20, the first session of TPE was managed using FFP. The volume of FFP used in the exchange was defined by the formula: Plasma volume = (0.065 × weight (kg)) × (1-hematocrit). A second session was needed (Table 2). No incident or complication during or after the plasmapheresis was recorded. Treatment with potassium iodide, propranolol, and prednisolone was discontinued afterward. After maintaining a clinical and biochemical euthyroid state, she underwent a total thyroidectomy.

### Case 3

A 29-year-old African woman was admitted to the Endocrinology Department for thyrotoxicosis with minor side effects (hives) to antithyroid drugs. She has been followed up with for Grave’s disease for 5 years, initially put on carbimazole 40 mg and propranolol 20 mg daily. The course was marked by the occurrence of urticarial lesions, leading to the switch from carbimazole to benzyl-thiouracil. The dose was gradually increased to 150 mg three times daily. However, her thyroid hormones remained dangerously elevated.

Upon physical examination, the heart rate was regular at 88 bpm, the BP was at 120/70 mm Hg, the respiratory rate was at 19 breaths/minute, and the SaO_2_ was 100%. The patient also reported episodes of palpitation and diarrhea. Cervical palpation found a thyroid hypertrophy that was responsible for dysphagia. We also noted bilateral proptosis and urticarial lesions.

Blood tests confirmed thyrotoxicosis without abnormalities in the liver or cardiac enzymes. On complete blood counts, we found microcytic anemia that needed venous iron infusion (Table 3). ECG and chest X-ray were normal. The transthoracic echocardiogram was normal, while cervical ultrasound showed a regular goiter of 30 ml in volume.

**Table 3:** Laboratory workup during the course of the patient’s illness (patient 3)

Tests	Day 1*	Day 2*	Day 4**	Day 8*	Day 9*	Day 10*	Day 11*	Day 12*	Day 13*	Normal range
TSHus (mUI/l)	0.01									0.25–4.2
FT4 (pmol/l)	56.9	58.5	50.3	42.4	32.6	29.31	25.7	23.05	22.5	12–22
WBC (µ/l)	4830	5590	4490	6810	6790	6910	6300	7210	7230	7000–10,000
ANC (µ/l)	2080	4100	1980	3650	2970	3360	3450	4060	4107	3000–5000
AST (UI/L)	16	24	24	23	16	19	16	18	18	5–45
ALT (UI/l)	23	27	33	30	29	26	24	24	25	5–35
CRP (mg/l)	11.7	5.3	2.44	10	8.67	11.24	8.3	6.2	4	0–5
Calcemia (mg/l)	87	85	86	90	96	93	89	89	89	84–105
PT (%)			100	100	100					70–100

*WBC* white blood cells, *ANC* absolute neutrophils count, *PT* prothrombin time, *AST* aspartate aminotransferase, *ALT* alanine aminotransferase, *CRP* C-reactive protein, *TSH* thyroid-stimulating hormone, *TSHus* ultrasensitive TSH assay, *FT4* free thyroxine

*Days of TPE

**Iodide potassium introduction

Benzyl-thiouracil was discontinued upon admission for ineffectiveness and urticaria. The patient was then started on propranolol 40 mg three times a day, cholestyramine 4 g three times a day, and prednisone 60 mg daily. Iodide of potassium was administrated later.

**Fig. 1:**
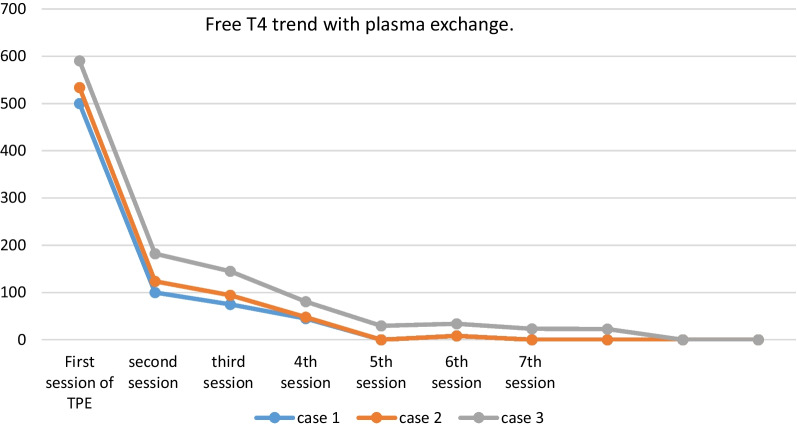
Evolution of FT4 after plasma exchange in the three patients

On the 11th day of admission, the first session of TPE was accomplished using FFP with an exchange volume of 3750 ml for each session. The patient required six more sessions of plasmapheresis to achieve clinical and biological euthyroidism (Fig. 1). Then, she underwent a total thyroidectomy without complications.

## Discussion

Thyrotoxicosis is a condition resulting from inappropriate excessive circulating thyroid hormone concentrations. A subtype of thyrotoxicosis, hyperthyroidism, specifically refers to excessive synthesis and secretion of thyroid hormones by the thyroid gland [10]. The most common cause is Graves’ disease (GD), which accounts for 80% of cases, followed by nodular thyroid goiter and thyroiditis [11].

Thyroid storm is a severe life-threatening exacerbation of thyrotoxicosis, characterized by the dysfunction of the thermoregulatory, central nervous, digestive, and cardiovascular systems [7]. Treatment strategies of this rare condition include thionamides, potassium iodide, bile acid sequestrants, glucocorticoids, and beta-blockers [1, 8]. Recently, therapeutic plasma exchange is considered more and more as an additional therapeutic option for the management of thyrotoxic patients. In 1970, Ashkar *et al.* [12] used this method for the first time in three patients with thyroid storm.

Plasmapheresis is based on extracorporeal separation of plasma from the blood. Plasma is separated from the cellular components of the blood using centrifugation techniques and discarded. The cellular components are then returned to the patient along with replacement fluids such as fresh frozen plasma, albumin, and crystalloids[13]. TPE needs to be performed by an experienced medical team and used carefully for appropriate indications [14].

There exists no clear consensus concerning its practice in hyperthyroidism without thyroid storm [7, 15]. Indeed, TPE can be performed in thyrotoxic patients with severe symptoms and rapid clinical deterioration, failure or adverse effects of conventional therapy, and obviously, multisystem organ failure[13]. In the latest guidelines of the American Apheresis Association (ASFA), thyroid storm is designated as a Category IIc and Category III recommendation for TPE [16]. ASFA recommend that TPE sessions should be executed by multidisciplinary trained medical team, as early as possible and repeated every 24 hours to every 3 days until clinical improvement [17].

To our best of knowledge, we report the first experience of plasmapheresis for thyrotoxicosis in our country. The three patients had Graves’ disease, and plasmapheresis was indicated for drug side effects (Case 2), thyroid storm complicated by heart failure (Case 1), and drug ineffectiveness (Case 3). Yildirim Simsir *et al.* [18] reported the largest series of TPE for thyrotoxicosis. In 46 patients, the most common etiology was Graves’ disease (87%), followed by amiodarone-induced thyrotoxicosis (8,7%), and toxic multinodular goiter (4,3%). In accordance to our cases, plasmapheresis indications were drug side effects (45.7%), drug ineffectiveness (19%), and thyroid storm (6%) [5].

The number of apheresis sessions in our cases was variable between two to seven sessions with an interval of 24 hours in accordance with the series of Keklik *et al.* [19] and Yildirim *et al.* [18] where the same interval between TPE sessions was respected. Yildirim *et al.* [18] reported a mean of four apheresis sessions [interquartile range (IQR): 3–7] in patients with Graves’ disease, and a mean of three sessions (IQR: 1–7) in patients with non-Graves’ thyrotoxicosis. There was no statistically significant difference between the two groups in terms of the number of sessions (*p* = 0.70) according to the cause of thyrotoxicosis. In the series of Keklik *et al.* [22], apheresis was applied with a mean of four times (minimum two, maximum nine). An average of 3.4 times (minimum 1, maximum 17) were performed in the study of Ezer *et al.* [20]. There is no clear recommendation of number of sessions of TPE. The ASFA recommends continuing sessions until clinical improvement, if there is no adverse effect.[17].

Plasmapheresis is not completely innocuous. Indeed, the incidence of serious and life-threatening complications of TPE is around 0.025–4.75% [21]. Transfusion reactions, citrate-related hypocalcaemia, coagulopathy/embolism, and anaphylactic reaction are the most frequent reported side effects. These complications are often catheter-related, and can easily be ruled out by an experienced medical staff. Death is rarely reported and is usually due to the primary disease [22]. In our series, there were no complications during or after the plasmapheresis sessions, especially those related to catheters (infection, thrombosis). In the study of Yildirim *et al.* [18], complications occurred in 6.5%, including catheter infection in two patients, and deep vein thrombosis in a pregnant patient. Another study reported hypotension, citrate-related hypocalcemia, and tachyarrhythmia in the course of myocardial infarction [19]. Particularly, our cases maintained normal calcium levels before and after the apheresis sessions. This finding is related to the use of heparin instead of citrate in our patients. This technique has recently been used in our facility, and demonstrated its efficacy in managing thyrotoxicosis in our patients. The second and third patients remained well until total thyroidectomy was performed. Unfortunately, the first patient died after refusing surgical treatment and discontinuing all medications against medical advice.

Our paper sheds light on the effectiveness of TPE in the management of refractory, severe thyrotoxicosis. The limiting factor of our case series report is the small number of patients. Our results/conclusions would be even stronger with a larger number of patients. We encourage consideration of this new approach as an effective treatment for patients with complicated or severe, refractory thyrotoxicosis who cannot tolerate, or do not respond to, standard treatment including thfionamides.

## Conclusion

In light of the literature and based on our experience, we conclude that TPE is an effective alternate therapeutic option in refractory severe thyrotoxicosis to prepare patients for surgical treatment. It is an appropriate treatment technique to obtain normal thyroid function rapidly and consistently. Thus, TPE should be included in the treatment algorithm for refractory cases or severe/complicated thyrotoxicosis. Therapeutic plasmapheresis should be optimally performed in tertiary centers by an experienced medical team to ensure safety and efficiency.

## Data Availability

Not applicable.

## Abbreviations

GD: Graves’ disease
TMNG: Toxic multinodular goiter
TN: Toxic nodules
T4: Thyroxin
T3: Triodothyronine
TPE: Therapeutic plasma exchange
FFP: Fresh frozen plasma
ASFA: American Society for Apheresis
ER: Emergency room
BP: Blood pressure
ECG: Electrocardiogram
TSH: Thyroid-stimulating hormone
ICU: Intensive care unit
IQR: Interquartile range
